# Proteomic Profiling of Cranial (Superior) Cervical Ganglia Reveals Beta-Amyloid and Ubiquitin Proteasome System Perturbations in an Equine Multiple System Neuropathy*[Fn FN2]

**DOI:** 10.1074/mcp.M115.054635

**Published:** 2015-09-13

**Authors:** Bruce C. McGorum, R. Scott Pirie, Samantha L. Eaton, John A. Keen, Elizabeth M. Cumyn, Danielle M. Arnott, Wenzhang Chen, Douglas J. Lamont, Laura C. Graham, Maica Llavero Hurtado, Alan Pemberton, Thomas M. Wishart

**Affiliations:** From the ^‡^Veterinary Clinical Sciences and; §Division of Neurobiology, The Roslin Institute and Royal (Dick) School of Veterinary Studies, University of Edinburgh, UK;; ^¶^FingerPrints: Proteomics Facility, School of Life Sciences, University of Dundee, Dundee, UK;; ^‖^ Euan MacDonald Centre for Motor Neuron Disease Research, University of Edinburgh, Edinburgh, UK

## Abstract

Equine grass sickness (EGS) is an acute, predominantly fatal, multiple system neuropathy of grazing horses with reported incidence rates of ∼2%. An apparently identical disease occurs in multiple species, including but not limited to cats, dogs, and rabbits. Although the precise etiology remains unclear, ultrastructural findings have suggested that the primary lesion lies in the glycoprotein biosynthetic pathway of specific neuronal populations. The goal of this study was therefore to identify the molecular processes underpinning neurodegeneration in EGS. Here, we use a bottom-up approach beginning with the application of modern proteomic tools to the analysis of cranial (superior) cervical ganglion (CCG, a consistently affected tissue) from EGS-affected patients and appropriate control cases postmortem. In what appears to be the proteomic application of modern proteomic tools to equine neuronal tissues and/or to an inherent neurodegenerative disease of large animals (not a model of human disease), we identified 2,311 proteins in CCG extracts, with 320 proteins increased and 186 decreased by greater than 20% relative to controls. Further examination of selected proteomic candidates by quantitative fluorescent Western blotting (QFWB) and subcellular expression profiling by immunohistochemistry highlighted a previously unreported dysregulation in proteins commonly associated with protein misfolding/aggregation responses seen in a myriad of human neurodegenerative conditions, including but not limited to amyloid precursor protein (APP), microtubule associated protein (Tau), and multiple components of the ubiquitin proteasome system (UPS). Differentially expressed proteins eligible for *in silico* pathway analysis clustered predominantly into the following biofunctions: (1) diseases and disorders, including; neurological disease and skeletal and muscular disorders and (2) molecular and cellular functions, including cellular assembly and organization, cell-to-cell signaling and interaction (including epinephrine, dopamine, and adrenergic signaling and receptor function), and small molecule biochemistry. Interestingly, while the biofunctions identified in this study may represent pathways underpinning EGS-induced neurodegeneration, this is also the first demonstration of potential molecular conservation (including previously unreported dysregulation of the UPS and APP) spanning the degenerative cascades from an apparently unrelated condition of large animals, to small animal models with altered neuronal vulnerability, and human neurological conditions. Importantly, this study highlights the feasibility and benefits of applying modern proteomic techniques to veterinary investigations of neurodegenerative processes in diseases of large animals.

Equine grass sickness (EGS, or equine dysautonomia) is a predominantly fatal, rapid multiple system neuropathy of grazing horses with reported incidence rates of 2.1–2.3% (reviewed by (1, 2)). An apparently identical disease occurs in cats, dogs, hares, rabbits, llamas, and possibly sheep (3–9). EGS is associated with chromatolysis of sympathetic and parasympathetic postsynaptic neurons, particularly in the enteric nervous system, as well as autonomic presynaptic and somatic lower motor neurons in the brainstem and spinal cord (10). EGS is subdivided into acute, subacute, and chronic forms according to the severity of clinical signs that largely reflect enteric and autonomic neurodegeneration, including dysphagia, generalized ileus, sweating, salivation, ptosis, rhinitis sicca, and tachycardia. While the etiology of EGS remains unknown, some evidence supports it being a toxic infection with *Clostridium botulinum* type C or D (11, 12). Ultrastructural studies suggest that the lesion in EGS primarily involves the glycoprotein biosynthetic pathway of specific neurons since the rough endoplasmic reticulum and Golgi complexes are consistently affected, while other organelles, including mitochondria, appear relatively normal (13). However, while the ultrastructural and cellular appearance of affected neurons has been studied extensively, little is known about the molecular mechanisms that contribute to neurodegeneration.

The overarching aim of this study was therefore to identify the molecular processes underpinning neurodegeneration in EGS using a bottom-up approach beginning with the application of modern proteomic tools to the analysis of cranial (superior) cervical ganglion (CCG, a consistently affected tissue) from EGS-affected patients and appropriate control cases postmortem. The cranial (superior) cervical ganglion (CCG), which supplies sympathetic innervation to the head and neck, was selected because chromatolysis of a high proportion of CCG neurons is a consistent feature of EGS (Fig. 1 and Supplemental Fig. 1 (14)). Here, proteomic analysis was carried out using isobaric tag for relative and absolute quantitation (iTRAQ) tools, which are now well established in small animal models of human neurodegenerative conditions but which are not routinely utilized in large animal models or large animal intrinsic conditions. This proteomic analysis was coupled with quantitative fluorescent Western blotting (QFWB), immunohistochemistry (IHC), and *in silico* based techniques in an attempt to identify the molecular pathways and processes that may be contributing to neurodegeneration in EGS. Here, we report widespread changes in the CCG of EGS horses, including significant disruption to a broad range of functional pathways clustering around candidates commonly associated with protein misfolding/aggregation responses in human neurodegenerative conditions.

**Fig. 1.**
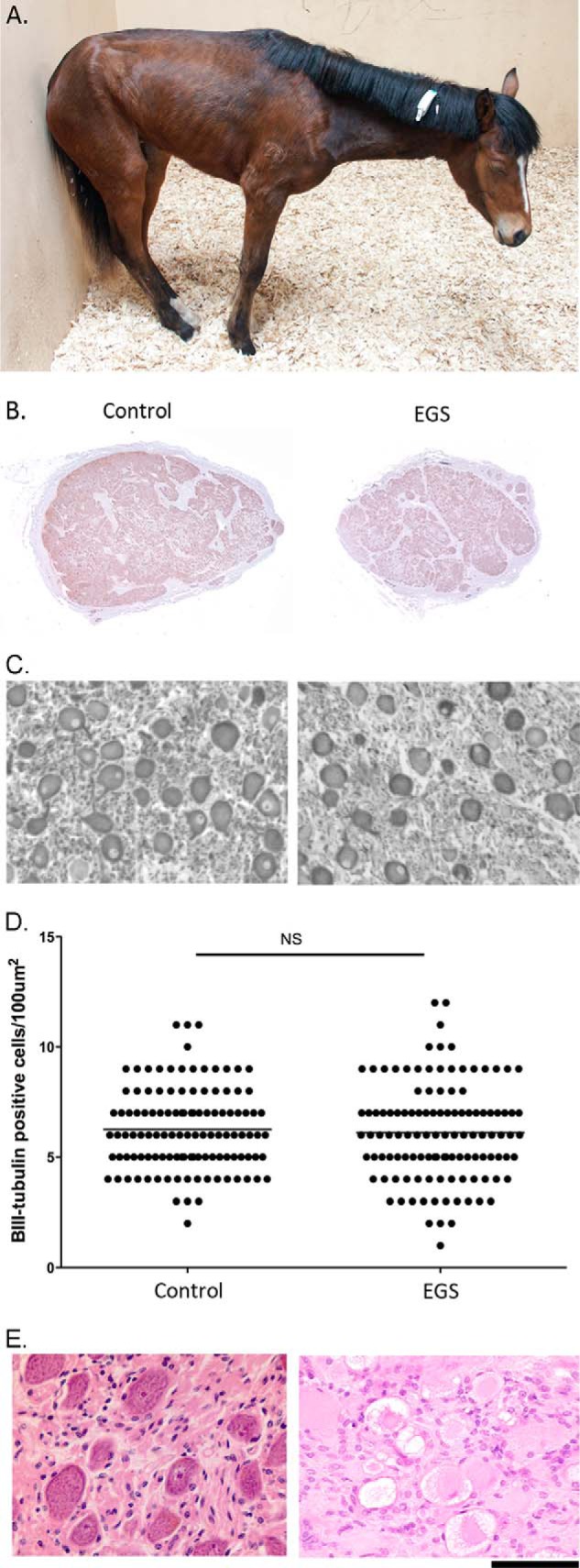
**Equine grass sickness is a predominantly fatal, acute multiple system neuropathy of grazing horses.** (*A*) Example photograph of a horse exhibiting typical appearance associated with chronic EGS. There is ptosis, and generalized muscle weakness as evidenced by the base narrow stance, low head and neck carriage, and leaning against a wall for support. Generalized muscle atrophy and reduced abdominal volume are also evident. (*B*) Example BIII-tubulin-stained sections from cranial cervical ganglia (CCG), which are known to exhibit neuronal perturbations in this disease. The visible puncta are BIII positive neurons. (*C*) High power micrographs stained CCG sections from *B* showing BIII positive neuronal profiles. (*D*) Quantification of BIII positive neurons demonstrates that there is still equivalent neuronal density in ganglia at terminal stages of the disease (control 6.25 ± 0.12, EGS 6.10 ± 0.20 cells per 100 μm^2^, mean ± Standard Error (S.E) *n* = 4 cases per condition, *n* = 116 grids measured. See Materials and Methods for more information). (*E*) Example H and E stained sections from control and EGS-affected CCG demonstrates that while the neuronal density may be similar, many of the neurons exhibit chromatolysis. Scale bar = 0.75ft (*A*), 0.5 cm (B), 35 μm (*C*), 100 μm (*E*).

This study therefore represents the first application of modern proteomic tools to equine neuronal tissues and/or to an inherent neurodegenerative disease of large animals (not a model of human disease). It is also the first to demonstrate correlation and conservation spanning the degenerative molecular cascades from an apparently unrelated condition of large animals to small animal models with altered neuronal vulnerability and a range of human neurological conditions from childhood neurodegenerative conditions such as spinal muscular atrophy through to diseases associated with advancing age such as Alzheimer's. Finally, this study highlights the feasibility and benefits of applying differential proteomics techniques to the investigation of the neurodegenerative processes in diseases of large animals.

## MATERIALS AND METHODS

In accordance with MCP guidelines, more detailed discussion of experimental design and potential limitations can be found in the accompanying Supplementary Discussion file. General methodology is provided below.

### 

#### 

##### Ethics Statement

Tissue samples were collected at necropsy from horses that were euthanized on humane grounds, with the horse owners' consent. The study was approved by the local ethics committee.

##### Collection of Ganglia

For proteomics and quantitative fluorescent Western blotting, CCG were collected from six EGS (median age 6 years, range 3–17) and six control (14, 6–30 years) mixed-breed and mixed-gender horses within 60 min of euthanasia by administration of barbiturates (Table I). CCG were selected because chromatolysis of a high proportion of CCG neurons is a consistent feature of EGS (see Fig. 1) (14). The heterogeneity in breed, sex, and age of EGS horses used in the study reflects the spectrum of horses affected by this spontaneous neurodegenerative disease during the study period. EGS horses comprised three acute and three subacute cases, as categorized by McGorum and Kirk (15). The grouping of acute and subacute cases for proteomic analysis was carried out as there are only minor differences in clinical features and pathology of these phenotypes. The main difference is the length of disease process following diagnosis. All of the samples are from post mortem terminal patients and the anatomical and cellular hallmarks are consistent at end stage regardless of case type. Moreover, we can demonstrate that neuronal density does not differ between acute and subacute cases (Supplemental Fig. 1). Due to these considerations and the fact that the initiating insult remains unproven, molecular analyses are currently performed on pooled samples as an attempt to reduce “noise” due to interanimal variability through factors such as individual disease response, age, and breed, among other considerations. EGS was confirmed in all cases by necropsy, including histopathological examination of autonomic ganglia (16). Controls were euthanized on humane grounds for reasons other than neurological disease. Immediately after collection, CCG were rapidly frozen by immersion in dry ice pellets and stored at −80 °C. For immunohistochemistry, CCG were collected from six EGS (median age 8 years, range 2–20) and six control (median age 11, 6–15 years) mixed-breed and mixed-gender horses as described above (Table I) and fixed in 10% neutral buffered formalin and embedded in paraffin wax.

**Table I:**
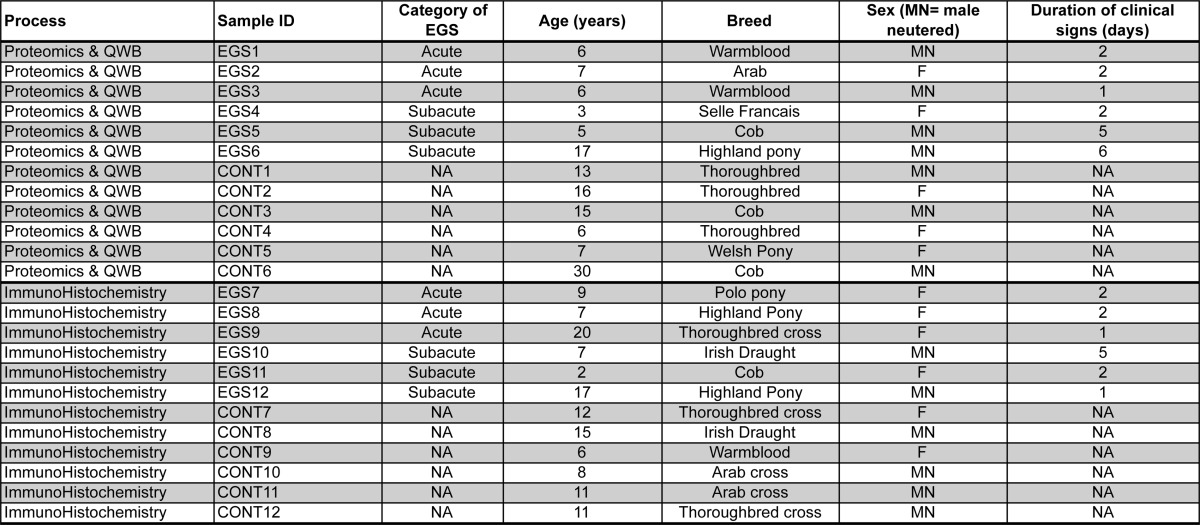
Subject information

##### Protein Extraction

Ganglia were partially thawed, the outer fascia removed by dissection, and a portion macerated with a scalpel before partial homogenization in either radioimmune precipitation assay buffer with protease inhibitor mixture (Roche) for QFWB (see below) or iTRAQ extraction buffer containing 6 m Urea, 2 m thiourea, 2% CHAPS, 0.5% SDS, and protease inhibitor mixture (Roche) for proteomic processing (see below). Samples were pooled by condition and manually homogenized in a dounce glass homogenizer. Homogenized samples were sonicated in a cup style sonicator six times for 15 s at power level 7.5 with vortexing for 30 s between each round of sonication. Samples were left on ice for 10 min before being revortexed then centrifuged at 20,000 *g* for 30 min at 4 °C. The resulting pellet containing proteins insoluble when processed in this manner was stored at −80 °C, and the supernatant was transferred to a fresh 1.5 ml tube to be processed for iTRAQ labeling as previously described (17–20).

##### iTRAQ Proteomic Analysis

Protein was extracted in iTRAQ extraction buffer (6 m Urea, 2 m thiourea, 2% CHAPS, 0.5% SDS, and protease inhibitor mixture (Roche, Burgess Hill, UK), (pH 7.4)) before acetone precipitation and labeling for iTRAQ analysis. The Mass spectrometry proteomic data have been deposited to the ProteomeXchange consortium via the PRIDE partner repository with the dataset identifier PXD002956.

**Fig. 2.**
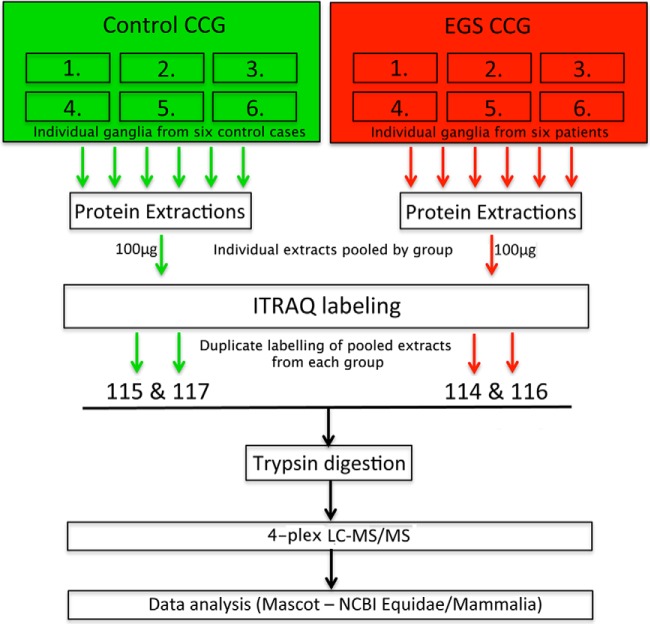
**Schematic of iTRAQ workflow.** Protein was extracted from six individual EGS and control horse CCG. Following separate extractions, small amounts of each were pooled by condition. Pooled samples were then labeled and run in duplicate to use all of the tags from an ITRAQ 4plex kit as previously described (17, 18, 19, 25). See Materials and Methods for further detail.

Protein extracts (*n* = 6 ganglia per group; see Fig. 2) were precipitated with −20 °C chilled acetone (1:4, v/v) and stored at −20 °C overnight. The precipitates were spun at 4 °C for 10 min then washed with an acetone:water mixture (4:1, v/v) twice prior to air drying. The pellets were then resuspended in iTRAQ sample buffer (25 μl 500 mm tetraethylammonium bromide, 1 μl denaturant (2% SDS), and 2 μl of reducing agent Tris(2-carboxyethyl)phophine (TCEP)). The samples were allowed to incubate for 1 h at 60 °C prior to protein estimation in triplicate (3 × 1 μl) by microBCA assay (Pierce, Paisley, UK). Samples were run in duplicate to utilize all four tags from the 4plex kit and increase peptide identification yield as previously described (17–20).

Each sample equivalent to 100ug was processed separately using the filter aided sample preparation (FASP) method prior to digestion with trypsin (sequencing grade, Roche). After digestion, the samples were dried using a SpeedVac concentrator and then resuspended in 25 μl of dissolution buffer as provided in the iTRAQ Reagents Multiplex kit (AB Sciex, Warrington, UK). The samples were then labeled, respectively, according to the protocol provided by the manufacturer (AB Sciex). The four labeled samples (Control-115 and 117, EGS-114 and 116) were then pooled together in equal proportions and subsequently dried using a SpeedVac concentrator. The pooled iTRAQ 4plex sample was then desalted using a homemade porous R2 ZipTip column and then fractionated by Strong Cation eXchange (SCX) using a Polysulfoethyl A column (2.1 × 200 mm, 5 μm, PolyLC) on a Ultimate U3000 (Dionex, Loughborough, UK) hplc system. The following buffer system was used Buffer A: 5 mm KH_2_PO_4_ in 20% CH3CN (pH 2.7) and buffer B: 500 mm NaCl in 5 mm KH_2_PO_4_ in 20% CH3CN (pH 2.7).The flow rate was set to 0.2 ml per minute with a linear gradient from 0 to 50% B over 25 min then a linear gradient from 50 to 100% B over 9 min.

Each fraction from the SCX fractionation was then dried using a SpeedVac concentrator and stored at −80 °C. Stored SCX fractions of the pooled iTRAQ 4plex sample were then resuspended in 10 μl of 5% formic acid, diluted to 1% formic acid, and then 15 μl aliquots injected onto an Ultimate RSLC nano UHPLC system coupled to a LTQ Orbitrap Velos Pro (Thermo Scientific, Loughborough, UK). The iTRAQ-labeled peptides were injected onto a trapping column (Acclaim PepMap 100, 100 μm × 2 cm, C18, 5 μm, nanoViper) and then separated using a 2 h linear gradient from 2–40% B (80% acetonitrile, 0.1% formic acid) on a separation column (Acclaim PepMap RSLC, 75 μm × 15 cm, C18, 2 μm, nanoViper) at a flow rate of 300 nl/min.

The mass spectrometry parameters were set as follows: Fourier transform mass spectrometry (FT-MS) (survey scan) resolution was set at 60,000; the 15 most-intense precursor ions were chosen for fragmentation by high energy collisional-induced dissociation (HCD); the precursor isolation window was set at 1.2 Da; and the ms/ms scan resolution was set to 7,500. Automatic gain control (AGC) values for FT-MS and FT-MS/MS were set at 1e6 and 5e4 ions, respectively. The maximum fill times for FT-MS and FT-MS/MS were set at 500 and 200 ms, respectively.

The raw data were extracted using Proteome Discoverer (Version 1.4.1, Thermo Scientific) for both quantitation and for identification by searching against the NCBI mammalia (database: NCBInr 20,121,028–21,171,493 sequences; 7,255,144,311 residues; taxonomy: mammalia (mammals) 1,173,629 sequences) and NCBI bacteria (database: NCBInr 20,130,811–31,351,517 sequences; 10,835,265,410 residues; taxonomy: bacteria (eubacteria) 20,989,102 sequences) databases using the Mascot Search Engine (Version 2.4.1, Matrix Science, London, UK). Search parameters included the following: enzyme: trypsin/P; fixed modifications (carbamidomethyl (C)), iTRAQ4plex (N-term); variable modifications: oxidation (M), dioxidation (M), acetyl (N-term), Gln->pyro-Glu (N-term Q), iTRAQ4plex (K), iTRAQ4plex (Y); peptide mass tolerance ± 10 ppm; fragment mass tolerance ± 0.06 Da; maximum of two miss-cleavages. Threshold score/expectation value for accepting individual spectra was based on Mascot ion score threshold (0.05) as the standard ion score threshold specifically calculated by Mascot for each database search. As an indication of identification certainty, the false discovery rate for peptide and protein matches above identity threshold were calculated by Peptide Validator at 1.0% (strict) and 5.0% (relaxed), respectively.

##### Validation of Candidate Proteins

Validation of altered expression levels for selected candidate proteins was carried out by quantitative fluorescent Western blotting using CCG protein extracts and by immunohistochemistry on CCG sections. See Table II for a list of antibodies and their compatibility with equine neural tissues.

**Table II:**
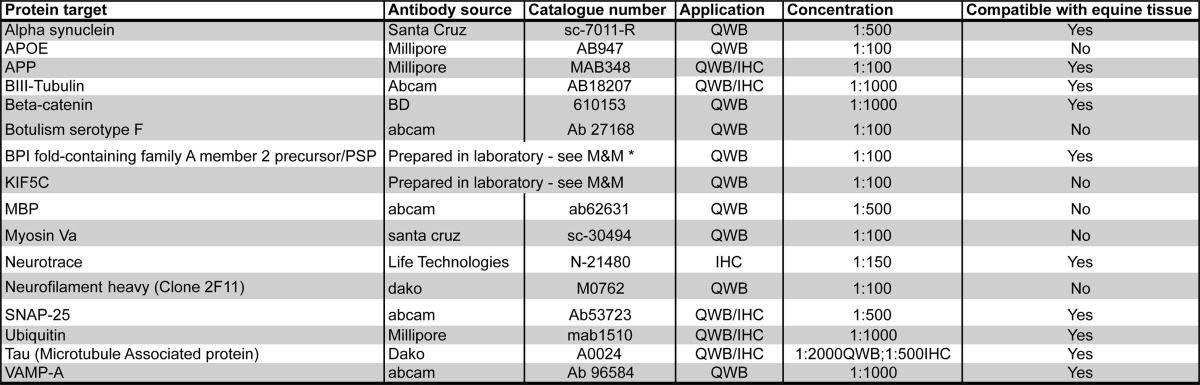
Antibody information

##### Quantitative Fluorescent Western Blotting (QFWB)

QFWB was carried out as previously described (21, 22). Briefly, 15 μg of CCG protein were separated by SDS-polyacrylamide gel electrophoresis on 4–12% precast NuPage BisTris gradient gels (Invitrogen, Paisley, UK) and then transferred to PVDF membrane using an iBLOT fast transfer device (Invitrogen). The membranes were then blocked using Odyssey blocking buffer (LICOR Biosciences, Cambridge, MA) and incubated with primary antibodies according to manufacturers' instructions (see Table II). Odyssey secondary antibodies were added according to manufacturers' instructions (goat anti-rabbit IRDye 680 and goat anti-mouse IRDye 800). Blots were imaged using an Odyssey Infrared Imaging System (LI-COR Biosciences). Scan resolution of the instrument ranges from 21 to 339 μm, and blots were imaged at 169 μm. Quantification was performed on single channels with the analysis software provided. Total protein stain gels, loaded in parallel with those used for membrane transfer, were used to ensure equivocal sample loading and were analyzed using the Odyssey Infrared Imaging System as previously described in (21, 22).

##### Immunohistochemistry (IHC)

Formalin-fixed, paraffin-wax-embedded CCGs were dewaxed and rehydrated. Antigen retrieval was performed by heating sections in 0.1 m citrate buffer (pH 6.0) for 15 min in a pressure cooker. The slides were then left to cool for 20 min. A commercial immunolabeling kit (DakoCytomation EnVision+ System-HRP; DAB K4001; DAKO, Ely, UK) was used according to the manufacturer's instructions. Slides were rinsed with Tris buffered saline containing 0.5% Tween (pH 7.5; TBST) and incubated with peroxidase blocking agent (Dako Real Peroxidase Blocker S2023) for 10 min. Slides were rinsed in TBST and incubated with murine monoclonal anti-human synaptophysin (Dako M0776) diluted 1 in 20 in TBST for 60 min at 25 °C. TBST replaced primary antibody for negative controls. Slides were rinsed and incubated with horseradish peroxidase-labeled polymer for 30 min then rinsed and incubated with substrate chromogen solution (Liquid DAB; ImmPact Dab SK4105; Vector Laboratories, Peterborough, UK) for 10 min. Slides were rinsed once in distilled water, counterstained with Harris's hematoxylin (1 min), dipped in Scott's tap water substitute, dehydrated, cleared using ethanol then xylene, and mounted under DPX. The intensity of labeling of CCG neurons was assessed blindly. IHC for B-APP, total Tau, and ubiquitin was carried out with antibodies detailed in Table II, for 2 h at room temperature, following microwave pretreatment in citrate buffer (pH 6), then labeled using the EnVision tracer system. For neuronal cell number assessment BIII-tubulin (Table II) IHC was carried out as described above and Neurotrace (Life-Technologies, Paisley, UK, Table II) was employed following manufacturer's instructions. Sections were visualized using a Nikon Eclipse E800 microscope, and whole sections were montaged by aligning approximately 80 10X images per ganglia section using Adobe Photoshop CS5.1 (V12.4) with the integrated Bridge module. BIII-tubulin and neurotrace-based neuronal counts were carried out in image j by overlaying a 10 × 10 square grid with each individual square measuring 100 μm^2^ (see Supplemental Fig. 1).

##### In Silico Protein Network Analysis

To obtain further insight into potential cellular pathways that may be modified as a result of protein changes identified in our experiments, the Ingenuity Pathways Analysis (IPA) application (Ingenuity Systems, Silicon Valley, CA) was used as previously described (17–20), (23). Pathway analysis has been demonstrated to highlight causative genes and mechanisms of disease in carefully conceived experimental paradigms (24). IPA dynamically generates networks of gene, protein, small molecule, drug, and disease associations on the basis of “hand-curated” data held in a proprietary database. More than 90% of the information in this database is “expert-curated” and is drawn from the full text of peer-reviewed journals. Less than 10% of interactions have been identified by techniques such as natural language processing. In the current analysis, candidate interactions were limited to experimentally observed interactions only but could be drawn from any source in the IPA database. Networks generated by IPA were limited in this study to ten networks comprising a maximum of 35 members per network. To enhance the explorative interpretation of data, networks are ranked according to a score calculated via a right-tailed Fisher's exact test. This test outputs a value that takes into account the original input gene or proteins of interest and the size of the network generated. The value enables the application to approximate how relevant the network is to the current analysis. It should be noted that this score does not indicate the biological relevance or quality of the network, which remains the prerogative of the analyst. Further information on the computational methods implemented in IPA can be obtained from Ingenuity Systems (http://www.ingenuity.com/).

##### Statistical Analysis

Unless otherwise stated, data were collected into Microsoft Excel spread sheets and analyzed using GraphPad Prism software. For all analyses *p* < .05 was considered to be significant. Individual statistical tests used are detailed in the results section or figure legends as appropriate.

## RESULTS

### 

#### 

##### Differential Proteomic Analysis of the Cranial (Superior) Cervical Ganglia (CCG) Reveals Widespread Proteome Disruption in Equine Grass Sickness (EGS)—A Multiple System Neuropathy

EGS-affected horse samples were collected immediately after horses were euthanized on humane grounds due to disease severity (median 2, range 1–6 days after the first onset of abnormal clinical signs) (Fig. 1 and Table I). At this stage of disease, histological examination commonly indicates that the majority of CCG neurons are undergoing acute neurodegeneration, and although neuronal density remains unaltered (Figs. 1*C* and 1*D* and Supplemental Fig. 1), there is likely to be a spectrum of stages of neurodegeneration present, ranging from neurons in the late stages of acute neurodegeneration to those with relatively normal morphology (see Fig. 1*E* and (14)).

**Fig. 3.**
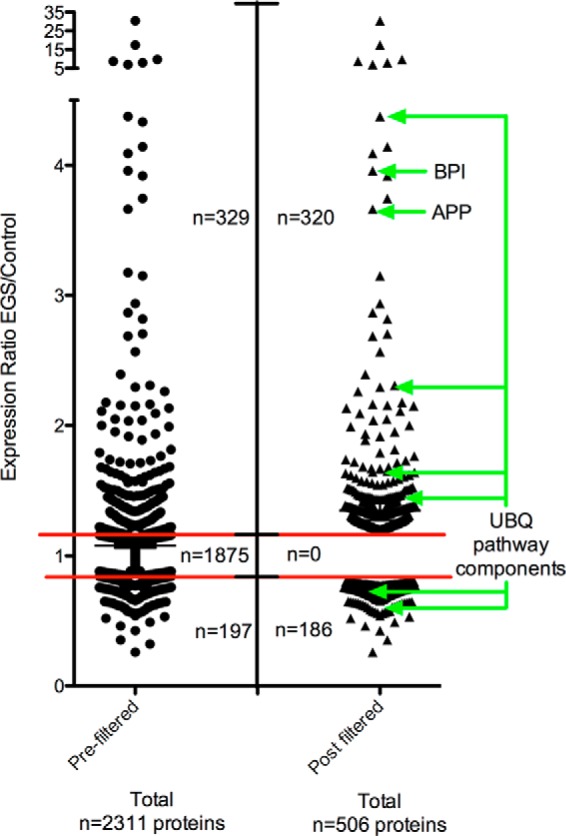
**Proteomic analysis identified 506 alterations in EGS CCG.** Scatter plot providing a graphical representation of the filtering process undertaken on the raw proteomics data in order to generate a final list of proteins altered in EGS compared with control CCG (26). Proteomic data were obtained by analyzing six control and six EGS horse CCG, and data are presented as expression ratios (EGS/control CCG) with each datum point representing an individual protein. The red bars indicate the 20% cutoff threshold for being up- or down-regulated in EGS ganglia compared with controls. Prefiltered (*LHS*) and postfiltered (*RHS*) data. Of the 2,311 proteins that were identified through iTRAQ analysis only 506 of those were considered differentially expressed following filtering. See Materials and Methods for further information on data filtering.

Using an iTRAQ quantitative proteomic approach, total protein extracts from six EGS patient CCG and six control samples were examined (See Fig. 2 and Materials and Methods section for details). 219,347 individual peptide sequences were submitted to Mascot (2.2) for identification using two separate databases (equidae and mammalia) due to the comparative lack of annotation within the equidae database. Mascot analysis returned identification of 2,311 proteins (Fig. 3, *left* data column and Supplementary Data File 1). Data were filtered to yield a final list of proteins altered in EGS CCG by only including those proteins identified by at least two unique peptides. Moreover, consistent with previously published methodologies to increase stringency in reporting, a minimum cutoff of 20% change *versus* controls was used as previously described (17–20, 23, 25, 26) (Fig. 3). This process resulted in the identification of 320 proteins that were considered to have increased levels in EGS ganglia and 186 with decreased expression (Fig. 3; Supplemental Tables 1 and 2). We therefore consider this refined list of 506 proteins to be representative of the molecular alterations induced by EGS neurodegenerative processes in CCGs. Due to the limited molecular investigations into EGS to date, the vast majority of proteins identified here with altered expression had not been previously associated with this disease.

To determine the veracity of the proteomic data, candidates were first selected for validation based on factors such as magnitude of change in the proteomic data set and potential relevance to neurodegenerative disease from the published literature. Candidates of immediate interest included BPI fold containing protein/parotid secretory protein (PSP—3.96 EGS/control ratio), amyloid beta precursor protein (APP—3.66 EGS/control ratio), and various components of the ubiquitin proteasome system (UPS; Fig. 3). PSP was of interest as a potential serum biomarker to aid diagnosis of EGS. APP was selected for validation due its repeated association with various human neurodegenerative conditions, including but not limited to Alzheimer's disease (27), prion diseases (28), tauopathies (29), murine neurodegenerative disease models, and as part of an acute phase response to neuronal injury (30). Altered expression of multiple components of the UPS was identified for follow up as specific ubiquitin cofactors such as ubiquitin carboxy-terminal hydrolase and PGP permeability glycoprotein (UCHL1/PGP) 9.5 have previously been reported in association with altered nasal mucosal and enteric innervation in EGS (31, 32) and various perturbations of the UPS have been implicated in multiple human neurodegenerative conditions, including but not limited to spinal muscular atrophy, Huntington's, Alzheimer's, and Parkinson's diseases (20, 33–36).

**Fig. 4.**
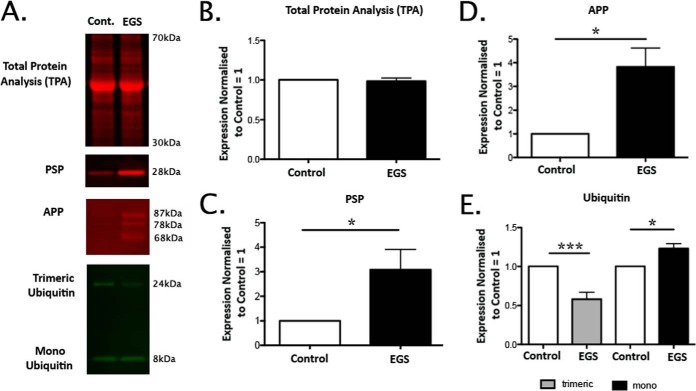
**Candidate validation of proteomic data by quantitative fluorescent Western blotting.** (*A*) Representative examples of bands used for quantification of total protein load, PSP, APP, and ubiquitin. (*B–E*) Graphical representation (bar charts; mean ± S.E.) of relative expression for total protein load and each candidate are given. Direction and magnitude of changes are consistent with iTRAQ data. All alterations were statistically significant (*n* = 4 horses per condition; unpaired two-tailed *t* test where *P0.05; **p < .01; ***p < .001).

Having selected candidates for validation, we began verifying the accuracy of the proteomic data by performing QFWB as previously described (17–21, 23). To confirm equivalent protein loading we carried out a total protein analysis with an Instant Blue stain (Fig. 4*B*; (21)). Total protein was used rather than conventional loading controls as these are frequently altered in tissue from neurodegenerative conditions and the EGS CCG proteomic data suggested that beta-actin (frequently used as a loading control) may be down regulated by 22% (0.78 EGC/control ratio; Supplemental Table 2). We can confirm (as demonstrated by QFWB) that both PSP and APP are indeed changed in the direction and to the approximate magnitude indicated by the proteomic data (3.08 ± 0.83, 3.82 ± 0.8—ratio EGS/control mean ± S.E., respectively; Figs. 4*B*-4*D*). Interestingly, it was possible to identify differential expression alterations in both mono- and multimeric ubiquitin, confirming that not only are specific UPS components altered but also that ubiquitin oligomerization profiles are different in EGS CCG. Here, we see an increase in “free” monomeric ubiquitin and a corresponding decrease in multimeric (-trimeric) ubiquitin (1.23 ± 0.06, 0.58 ± 0.09—ratio EGS/control mean ± S.E., respectively; Figs. 4*A* and 4*E*). This QFWB data therefore indicate that the proteomic analysis is likely to be an accurate reflection of the molecular alterations occurring in EGS neural tissue.

##### Cellular Expression and Distribution Profiles of PSP, APP, and Ubiquitin Are Altered in EGS CCG

**Fig. 5.**
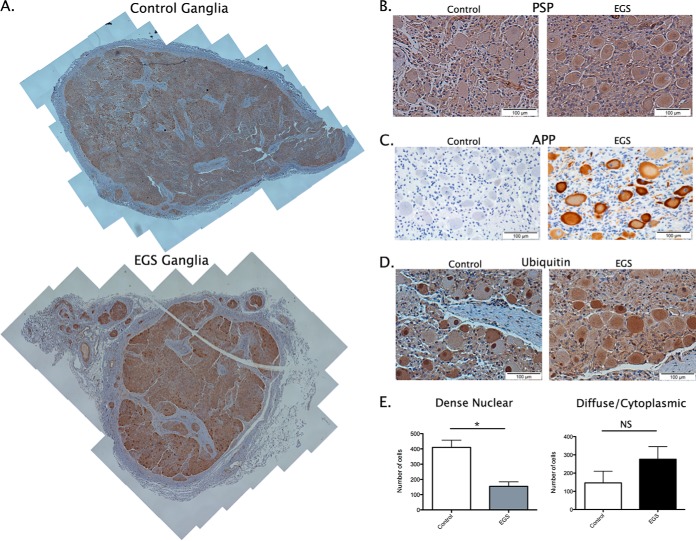
**Cellular expression of candidates further validates proteomic data confirming disruption of APP and the UPS.** (*A*) Example whole CCG section reconstruction from one control and one (acute) EGS ganglia. Each reconstruction comprises ∼80 images captured at 10X magnification. See methods for further information. (*B–D*) Representative images of DAB based immunohistochemistry to examine the cellular expression profiles of candidate proteins, including PSP, APP, and ubiquitin. (*E*) Quantification of ubiquitin expression demonstrates an increased trend in cells with diffuse cytoplasmic staining (88.8%± 47.4) and a decrease in dense nuclear staining of 62.3 ± 7.2% in EGS CCG compared with control CCG. (mean ± S.E.; *n* = 3 ganglia per condition (EGS = 2 acute and 1 subacute; 400–600 cells per ganglia) scale bar = 100 μm (*B–D*).

Given the status of proteins associated with misfolded protein responses in the pathogenesis of a broad range of neurodegenerative conditions in humans, we next wanted to extend the validation of our proteomic analysis to examine these candidates at the cellular level. While quantification by QFWB on whole tissue protein homogenate is a useful technique for determining relative protein expression levels, in this case, it only provides an indication of protein abundance irrespective of conformation or subcellular localization. Given the heterogenous cellular nature of the CCG (see Supplementary Discussion file) and the complex cellular functions of the proteins selected for validation by QFWB, we next sought to examine their expression profiles at the cellular level. Here, we carried out DAB-based IHC with whole plane analysis of reconstructed CCG. Each staining run and subsequent analysis comprises the examination of whole reconstructed sections (approximately 80 images at 10X magnification each—see Materials and Methods) from three EGS (two acute and one subacute) and three control ganglia (Fig. 5*A*). Having identified PSP by proteomic analysis and confirmed its alteration by QFWB, it appears from IHC that the increased expression is predominantly within cell bodies of EGS CCG (Figs. 3, 4*A*, 4*C*, and 5*B*). APP is a highly conserved protein (37), integral to cellular membranes and enriched in neurons (38). When we examined EGS sections for APP expression, there were obvious increases in cellular APP (Fig. 5*C*). Interestingly, the APP immunostaining is not purely membrane bound but appears as strong diffuse cytoplasmic staining with “rings” of APP denoting the membrane boundary of the cell. The staining intensity and subcellular distribution of APP suggests that the increase in APP expression reflects either increased protein synthesis and/or failure of APP transport from the perikaryon to the nerve terminus, perhaps correlating with previously reported defects in the Golgi apparatus and axonal transport (13).

Ubiquitin is a protein with complex roles within neurons. Upon further examination by QFWB (Figs. 4*A* and 4*E*) an increase in “free” mono ubiquitin (see Supplementary Tables; Fig. 3; (39)) and a resulting reduction in higher-order oligomers was seen. This is consistent with increased expression of deubiquitinating proteins such as members of the UCHL family (Supplemental Table 1). In neurons, such alterations result in an increase in diffuse ubiquitin staining (cytoplasmic) and a reduction in dense nuclear staining (higher-order oligomers) such as shown in EGS ubiquitin immunostained CCG sections relative to controls (Fig. 5*D*). Similar accumulations have previously been reported in TG22/UCHL1 overexpressing mice (39). Quantitative assessment of these sections demonstrates an increased number of cells with strong diffuse perikaryon staining (88.8%± 47.4; mean ± S.E. *n* = 3 ganglia per condition; 400–600 cells per ganglia) and a decrease in the appearance of dense nuclear accumulations 62.3 ± 7.2% (mean ± S.E. *n* = 3 ganglia per condition; 400–600 cells per ganglia) in EGS horses compared with controls. This information therefore serves to further verify the robust nature of the proteomic characterization of the molecular alterations taking place in EGS ganglia. Moreover, these data represent the first demonstration that the neurodegenerative processes in EGS share some molecular characteristics described in other neurodegenerative conditions, including humans and their murine models such as the accumulation and/or redistribution of proteins such as APP and ubiquitin.

##### In silico Analysis Highlights Evolutionarily Conserved Molecular Neurodegenerative Alterations and Higher-Order Candidate Clustering in EGS

Having confirmed the veracity of the proteomic data, we attempted to obtain further insights into potential molecular pathways and processes that may be modified by explorative *in silico* analysis using IPA software (see Materials and Methods). This application was used to identify direct and indirect molecular interactions, pathway associations, and functional assignments involving the filtered candidate list (Fig. 3, RHS; Supplemental Tables 1 and 2).

**Fig. 6.**
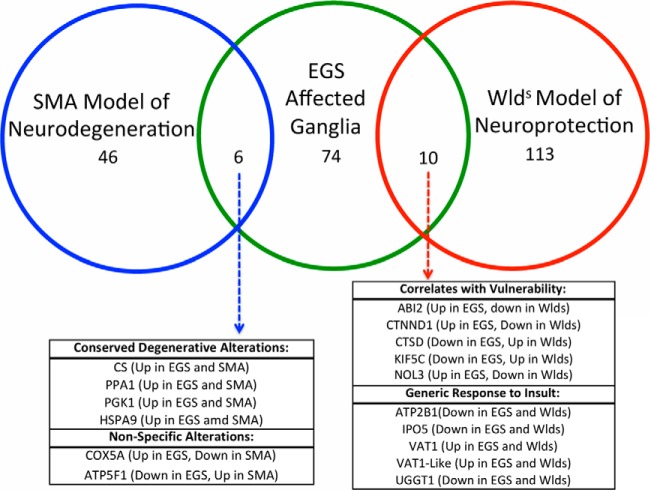
**Venny-based comparison of EGS protein alterations highlights molecular overlaps with published datasets from systems with altered neuronal stability.** Schematic representation of the overlaps between EGS proteomic data and that from murine models of neuroprotection (Wlds) and the human neurodegenerative disease spinal muscular atrophy (SMA). SMA is an inherited childhood neurodegenerative condition and data used in this alignment have previously been published in (17). Wlds represents a spontaneous mutation resulting in a neuroprotective phenotype. The dataset used for comparison here is taken at 2 days following a corticostriatal lesion in which Wlds tissues demonstrate no signs of neurodegenerative processes. The original data can be found in (18). 12% of SMA candidates and 8% of Wlds candidates are also found in the EGS analysis indicating overlap with other neurodegenerative processes at the molecular level (18% of viable EGS candidates). Interestingly, the data highlight candidates that may contribute to the degenerative responses (conserved in SMA and EGS), those that may be generic responses to a neurodegenerative insult (conserved in Wlds and EGS in direction), and those that may be regulatory in nature (opposite in Wlds and EGS).

Of the 506 proteins identified as altered in our proteomic comparison, those differentially expressed proteins that were adequately annotated to be eligible for *in silico* analysis, using the IPA database (Table III), clustered predominantly into the following higher-order biofunctions: (1) diseases and disorders, including neurological disease (32%), skeletal and muscular disorders (24%), and (2) molecular and cellular functions, including cellular assembly and organization (41%), cell-to-cell signaling and interaction (18%, including epinephrine, dopamine, and adrenergic signaling and receptor function), and small molecule biochemistry (25%). Interestingly, closer examination of the diseases and disorders biofunction suggests that up to 20% of the candidates have previously been reported in the published literature as being associated with specific neurodegenerative disease, including but not limited to Huntington's, Parkinson's, and motor neuron diseases (Table IV). To further explore the implication that common molecular correlates of neurodegenerative processes have been observed, we carried out a small-scale comparison of candidates identified here as being differentially expressed in EGS affected compared with control tissue, with existing published data from a mouse model of a neurodegenerative condition (SMA; See (18) for original published dataset) and a model of neuroprotection (Wlds; see (17) for original published dataset; Fig. 6). This comparison confirms that several candidates identified in EGS are also altered in both SMA-mediated neurodegenerative processes (12% of SMA candidates) and Wlds-mediated neuroprotective processes (8% of Wlds candidates). While this overlap in datasets is limited, it does yield interesting insights, *i.e.* those that may be contributing to the degenerative responses (conserved in SMA and EGS), those that may be generic responses to a neurodegenerative insult (conserved in Wlds and EGS in direction), and those that may be regulatory in nature (opposite in Wlds and EGS) (Fig. 6). For example, members of the catenin family have been implicated as drugable regulators of degenerative processes in disease (Supplemental Fig. 2; (20)) and members of the cathepsin family are known to instigate degenerative processes in lysosomal storage disorders (40). This information coupled with an examination of cellular processes highlighting an association with neuronal morphology and function (Table IV) suggests that many of the molecular processes occurring in EGS ganglia may be consistent with fundamental neurodegenerative processes, likely conserved across different conditions and species. These data, therefore, highlight the value of applying differential proteomics techniques to the comparative investigation of neurodegeneration in spontaneous human and animal diseases.

**Table III:**
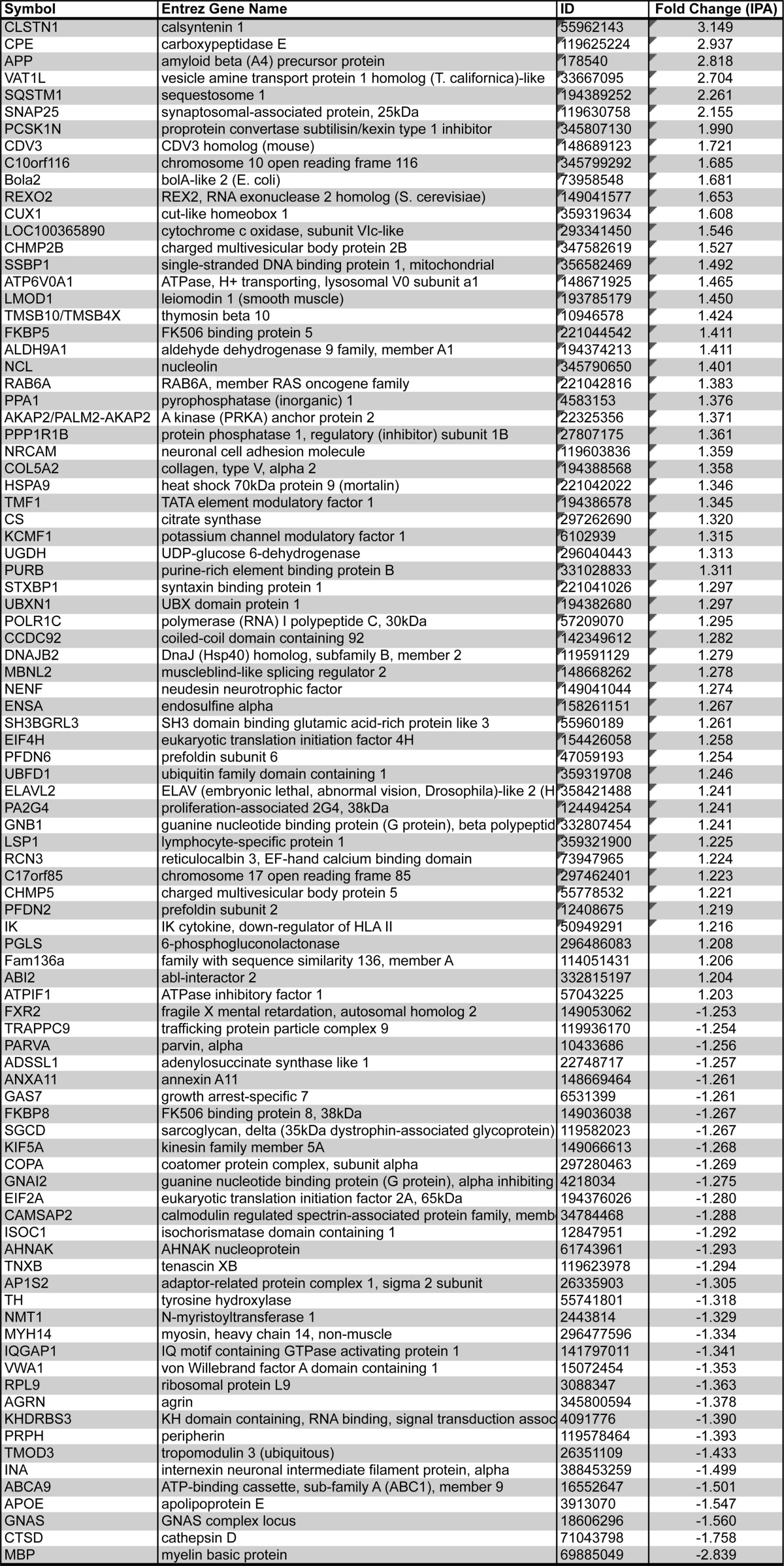
Proteins compatible with Ingenuity Pathway Analysis Database

**Table IV:**
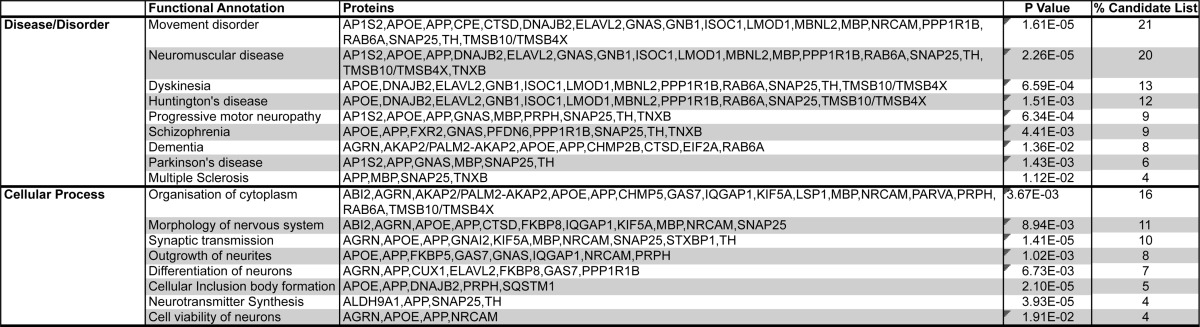
Ingenuity Pathway Analysis of Higher Order Functional Clustering

##### IPA Analysis of Multiprocess Interactions Highlights an APP-Centric Network

**Fig. 7.**
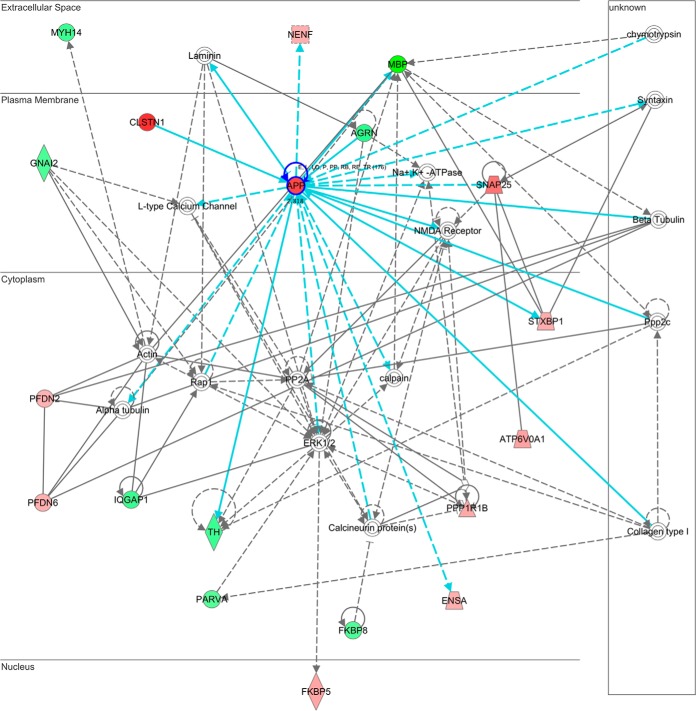
**Pathway analysis highlights** **APP is a central hub interacting with nearly 40% of candidate molecules in EGS.** This interaction network (comprising aspects of cell–cell signaling and interaction, nervous system development, and small molecule biochemistry) involves 38% of IPA viable candidates with modified expression in EGS. Proteins within this network identified as being modified in EGS CCG are indicated using *adjacent circles* (*red*, up-regulated in EGS; *green*, down-regulated in EGS; *clear*, absent from our dataset but integral to the network). *Solid connecting lines* indicate a direct interaction, and *dashed connecting lines* indicate an indirect interaction. *Blue connecting lines* represent the highlighted interactions of APP. All suggested indirect interactions were confirmed manually using Ingenuity Pathways Analysis software to identify published evidence indicating an interaction between the two proteins.

Coupled with the demonstration that the ubiquitin system and APP abundance and distribution are perturbed in EGS (Figs. 3, 4*A*, 4*D*, and 5*C*), IPA-based pathway analysis highlighted a network that appears likely to represent a molecular system whose perturbation may be actively contributing to EGS-induced neurodegenerative processes (Fig. 7). This network comprises functions associated with cell-to-cell signaling and interaction, nervous system development and function, and small molecule biochemistry (*n* = 38% of candidates from Table III; Fig. 7). In this network, APP is a central hub with both direct and indirect interactions encompassing a high proportion of the protein components (23/35 molecules, or 65%) with more than half of these originating from APP (14/23 molecules, or 61%). In combination with the molecular and cellular data provided in this study demonstrating a perturbation of the abundance and cellular localization of APP (Figs. 3, 4, and 5) and its existing high profile associations with a range of human neurodegenerative conditions (36–38), this, therefore, identifies for the first time APP as an attractive candidate as a potential regulator for the processes observed in EGS.

##### In Silico Analysis Predicts Upstream Regulators of EGS Identified Alterations

IPA software now provides the tools to predict potential upstream regulators of the functions that may be perturbed in the system of interest (24). These potential upstream regulators (transcriptional or translational) are correlative with the observed expression differentials in a specific subset of submitted data and may aid in the identification of cascade initiators. Table V lists the top five proposed upstream regulators ranked by *p* value. All of the five potential regulators highlighted by this analysis are interesting predictions for a number of reasons. They have all been implicated in human neurodegenerative conditions in which protein aggregation/misfolding is a pathophysiological hallmark (41–45). Tau is an interesting potential candidate because of its reported association with a range of human neurodegenerative conditions. Additionally, PP2A, which accounts for ∼70% of Tau phosphatase activity, also occupies a potential regulatory hub in Fig. 7. Other factors that can regulate neurodegeneration in their own right and degrade Tau are also decreased in EGS (*i.e.* cathepsin D, Table III). Here, we can confirm by QFWB that Tau is more abundant in EGS but only in the acute form (Supplemental Fig. 2). It is therefore likely that Tau alterations are consequential rather than causative. Prion protein has previously been considered as a potential initiator of the disease process in EGS, but this has never been proven, and prion protein was not present in our dataset. However, QFWB of CCG protein isolates from EGS and control horses suggests that there is no disease-associated prion protein (PrP^res^) present (Supplemental Fig. 3). Of the proposed upstream regulators, only APP appeared in the functional clustering/network analysis of the proteomic data. This further supports the data presented above and strengthens the suggestion that APP may be a key regulatory protein in EGS-related degenerative processes.

**Table V:**
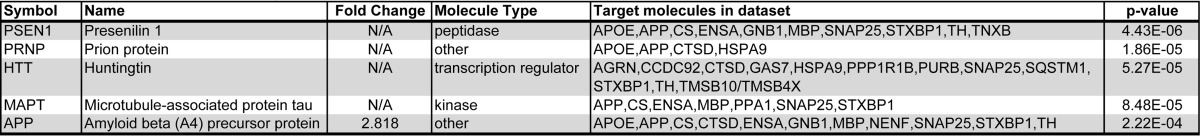
Ingenuity Pathway Analysis - Top Five Predicted Upstream Regulators

## DISCUSSION

### 

#### 

##### Patient Tissue Profiling Yields Insights into Molecular Processes that Must Be Considered in Accordance with Disease Staging

While studies of neurodegenerative diseases utilizing post-mortem patient tissues remain constrained by sample availability, the data presented here represent a combined molecular snapshot of late-stage disease processes from six horses with a spontaneous multiple system neuropathy (EGS) in comparison to six unaffected controls. Importantly, significant perturbations of the cellular proteome are evident even from analysis of a heterogeneous population of EGS animals (Table I), suggesting that many of the alterations identified are likely to be fundamental in the progression of this disease.

At the stage of disease when CCG samples were collected, histological examination indicates that CCG neurons are undergoing a spectrum of stages of neurodegeneration and that the majority are undergoing acute neurodegeneration. Consequently, the proteome of EGS CCG extracts will likely reflect the overall range of proteins originating from individual neurons at various stages of degeneration. Therefore, proteins representative of nonspecific neuronal death responses, as well as representatives of the processes initiating and regulating the neurodegenerative progression of EGS, will have been identified (see Fig. 5). The altered expression levels of distinct neural proteins documented in the current study may prove helpful not only for deepening our understanding of the pathophysiology of EGS but also for the future establishment of a comprehensive molecular signature of the disease.

To address the, albeit unlikely, possibility that the extracts from EGS CCG contained a protein neurotoxin derived from a causal bacterium or fungus, iTRAQ data were also run through NCBIs all species and bacterial databases. While Mascot analysis returned identification of 2,301 in the all species database (Supplementary Data File 1), it only identified 37 proteins in the bacterial database (Supplementary Data File 2). However, none of these were known toxins or were considered to be of relevance to the aetiopathogenesis of EGS. It is important to note that this does not preclude EGS being caused by a bacterial or fungal neurotoxic protein. It remains possible that the coverage of the proteome may be insufficient to facilitate toxin identification, a toxin may be present at concentrations below detection limits, or a toxin may not persist within the CCG of EGS horses.

##### Equine Proteomic Profiling Reveals Similarities to Other Neurodegenerative Conditions

Specific alterations in the EGS CCG proteome included multiple members of the ubiquitin proteasome system (UPS) (Fig. 3). Here, we identified many ubiquitin expression alterations, including a range of UCH family members, Ubas, and ubiquinone (Fig. 3, Supplemental Tables 1 and 2). Specific ubiquitin cofactors such as UCHL1/PGP 9.5 have previously been reported in association with altered innervation in nasal mucosal (31) and autonomic ganglia (32) in EGS. Ubiquitination is a key regulator of cellular homeostasis through its involvement in the management of protein misfolding, sequestration, degradation, and processing, including the processing of APP ((46); ubiquinone—Supplemental Table 2). Perturbations of the UPS have been implicated in various human neurodegenerative conditions, including; Alzheimer's, Huntington's, Parkinson's, and motor neuron diseases (20, 33–36). Moreover, through pathway analysis of the data generated in this study, we highlighted similarities and potentially conserved molecular processes between these prominent human neurodegenerative conditions and this equine multiple system neuropathy (Table IV, Fig. 5). Further discussion of specific candidates of specific interest to the neurodegenerative and equine clinical communities, which would detract from the focus of the manuscript, can be found in the accompanying supplementary discussion text (Supplementary Discussion File 1).

##### Analysis of Higher-Order Functional Clustering Yielded Several, Previously Unreported, Insights into the Processes Underpinning EGS Pathogenesis

For example, one of the cellular processes identified as being significantly altered in EGS was synaptic transmission (Table IV). The differentially expressed proteins associated with synaptic transmission, APOE, APP, SNAP25, and STXBP1, are also involved in regulating presynaptic terminal stability and synaptic vesicle fusion. Moreover, these proteins have also been reported in association with epilepsy, ataxia, and Parkinson's disease (47–51). It is therefore highly likely that dysregulation of these proteins contributes to neurodegeneration in EGS.

Additionally, it appears that the proteins identified by clustering analysis and proteins whose expression is consistent in other neurodegenerative diseases (*i.e.* citrate synthase—CS in SMA) or correlate with vulnerability status (*i.e.* cathepsin d—CTSD in Wlds; see Fig. 5) all share a common upstream regulator—APP. Upstream regulator analysis of the alterations identified in EGS highlighted a broad range of candidates associated with numerous human neurodegenerative diseases, many of which are characterized by protein misfolding and/or aberrant accumulation (Table V, Supplemental Figs. 2 and 3 (52–54)). In combination with confirmation of altered abundance (proteomics and QFWB), histological demonstration of mislocalization, and its identification as a central hub interacting with 65% of the members of a functional network (Fig. 7), APP therefore appears to be the most promising candidate upstream regulator of the alterations detailed in the current study. As such, APP is likely to be a regulator of EGS pathogenesis.

##### Summary

Reliable markers with potential modulatory ability are urgently required to improve both diagnostic and therapeutic efficacy, and these remain the key challenges for all neurodegenerative disease research. This is the first application of modern proteomic tools and *in silico* analytical techniques to equine neuronal tissues and/or to an inherent neurodegenerative disease of large animals (not a model of human disease). As such, it has facilitated the identification of more than 500 protein alterations in equine grass sickness cranial (superior) cervical ganglion, most of which had not been previously linked with EGS. This is also the first demonstration of correlation and conservation for neurodegenerative molecular cascades spanning from an apparently unrelated large animal neuropathy, small animal models of altered neuronal vulnerability, and a broad range of human neurological conditions. Finally, this study highlights the feasibility and benefits of applying differential proteomics techniques to the investigation of the neurodegenerative processes in diseases of large animals.

## Supplementary Material

Supplemental Data
