# Supplementary material for: Proteomic Profiling of Cranial (Superior) Cervical Ganglia Reveals Beta-Amyloid and Ubiquitin Proteasome System Perturbations in an Equine Multiple System Neuropathy
Source: Mol Cell Proteomics. 2015 Sep 13;14(11):3072–86. doi: 10.1074/mcp.M115.054635 (PMC4638047; doi:10.1074/mcp.M115.054635)
Supplement: Supplemental Data [file supp_14_11_3072__index.html]

Proteomic Profiling of Cranial (Superior) Cervical Ganglia Reveals Beta-Amyloid & Ubiquitin Proteasome System Perturbations in an Equine Multiple System Neuropathy — Proteomic Profiling of Cranial (Superior) Cervical Ganglia Reveals Beta-Amyloid and Ubiquitin Proteasome System Perturbations in an Equine Multiple System Neuropathy — Proteomic Characterization of an Equine Multiple System Neuropathy — Supplemental Data 

# Proteomic Profiling of Cranial (Superior) Cervical Ganglia Reveals Beta-Amyloid and Ubiquitin Proteasome System Perturbations in an Equine Multiple System Neuropathy

## Supplemental Data

- Supplementary data file 1: NCBI mammalia database analysis. - Supplementary data file 1: NCBI mammalia database analysis. Raw output
- Supplementary data file 2: NCBI bacterial database - Supplementary data file 2: NCBI bacterial database. Raw output
- Supplementary data file 3 EGS protein and peptide info - Supplementary data file 3 EGS protein and peptide info for MCP compliance
- Supplementary Table 1: Proteins Increased by greater than 20% in EGS (Min 2 unique peptides) - Supplementary Table 1: Proteins Increased by greater than 20% in EGS (Min 2 unique peptides)
- Supplementary Table 2: Proteins decreased by greater than 20% in EGS (Min 2 unique peptides) - Supplementary Table 2: Proteins decreased by greater than 20% in EGS (Min 2 unique peptides)
- McGorum et al\_Supplemantary Figure 1\_Neuron count variability - McGorum et al\_Supplemantary Figure 1\_Neuron count variability
- McGorum et al\_Supplemantary Figure 2\_protein variability - McGorum et al\_Supplemantary Figure 2\_protein variability
- McGorum et al\_Supplemantary Figure 3\_Prion - McGorum et al\_Supplemantary Figure 3\_Prion
- Supplementary discussion file - Supplementary discussion file. Document highlighting experimental design rational, limitations and results of interest to veterinary clinicians.
