# Supplementary figures and images for: Proteomic Profiling of Cranial (Superior) Cervical Ganglia Reveals Beta-Amyloid and Ubiquitin Proteasome System Perturbations in an Equine Multiple System Neuropathy
Source: Mol Cell Proteomics. 2015 Sep 13;14(11):3072–86. doi: 10.1074/mcp.M115.054635 (PMC4638047; doi:10.1074/mcp.M115.054635)

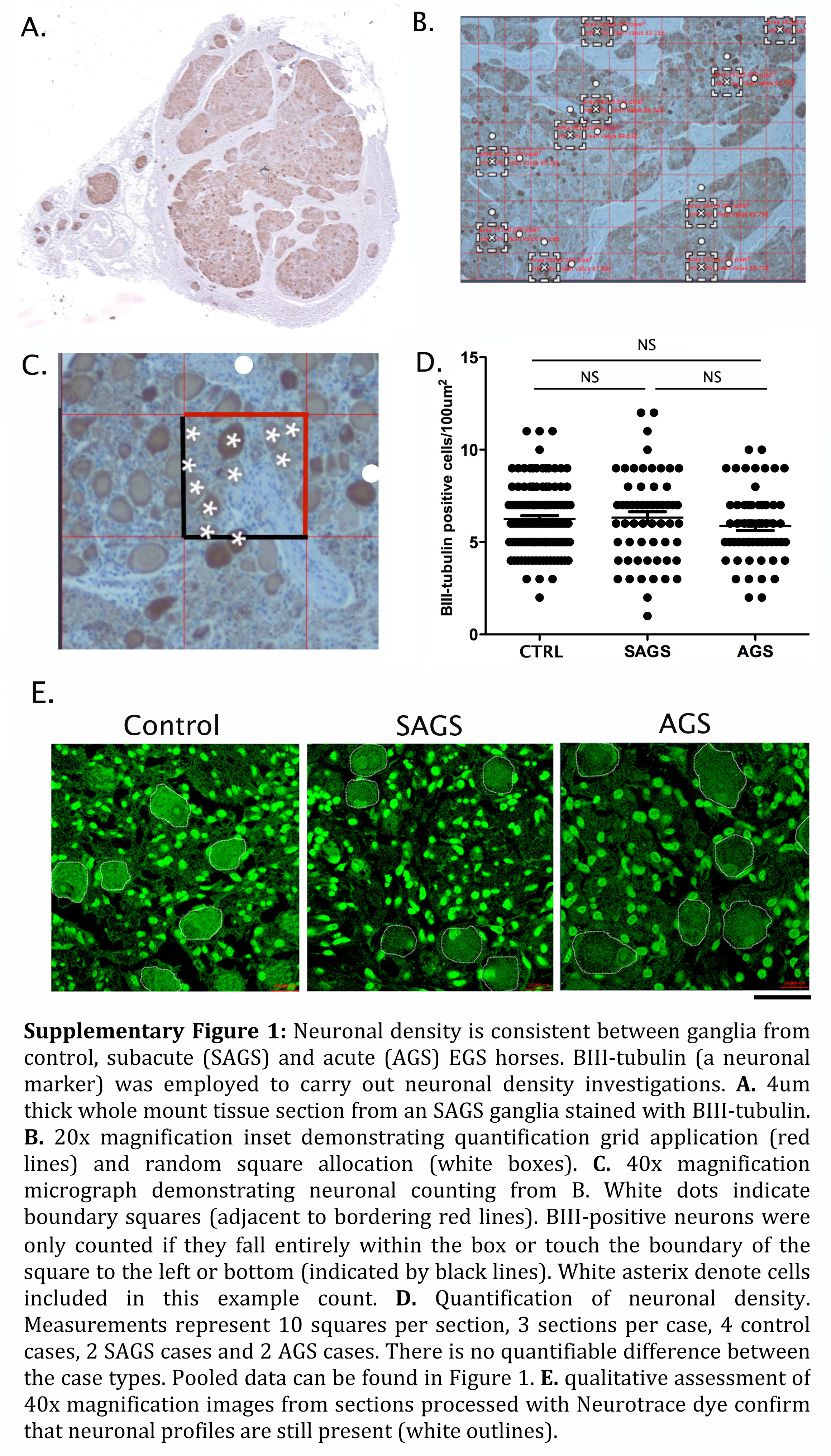

Supplement: Supplemental Data [file supp_M115.054635_mcp.M115.054635-6.jpg]

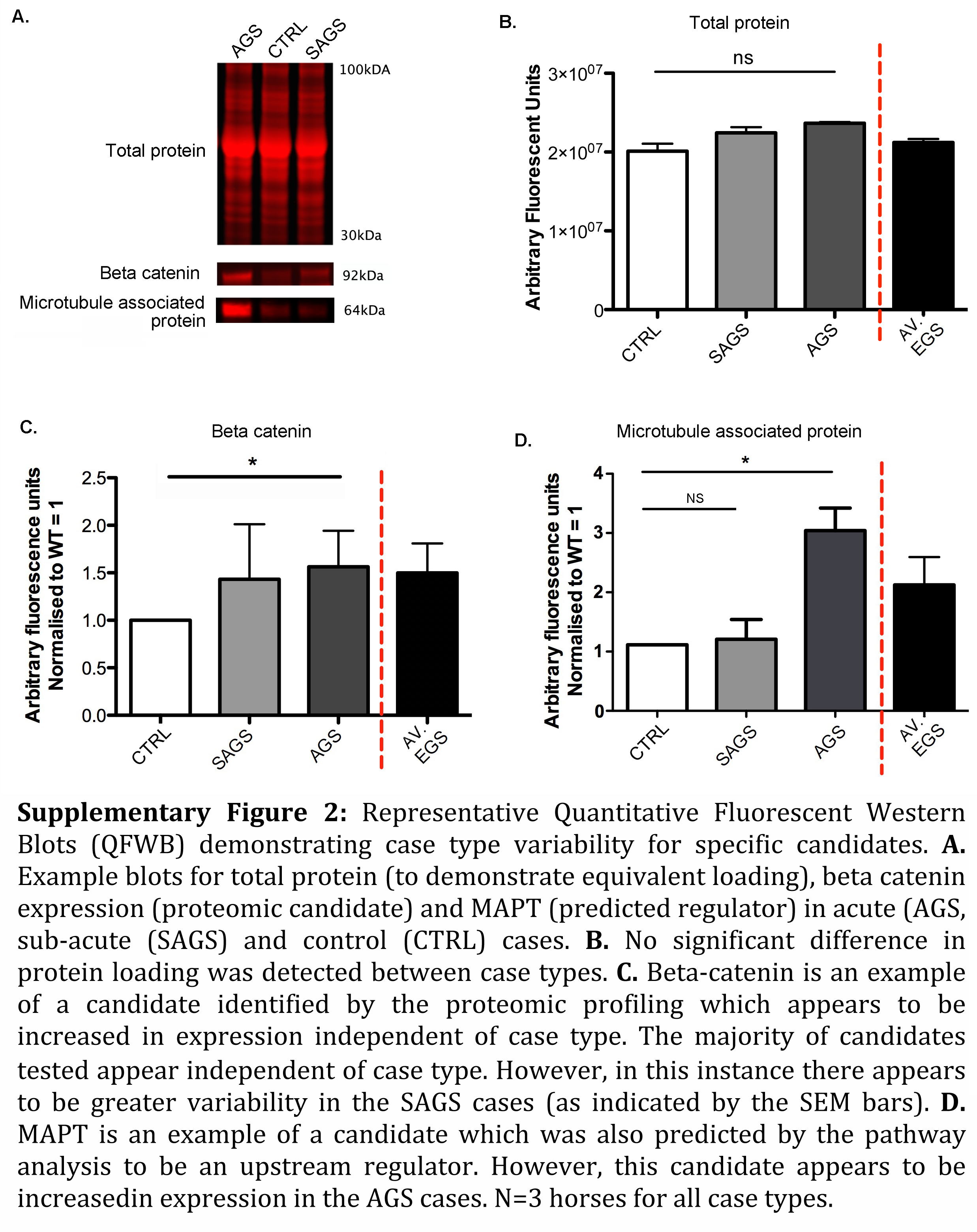

Supplement: Supplemental Data [file supp_M115.054635_mcp.M115.054635-7.jpg]

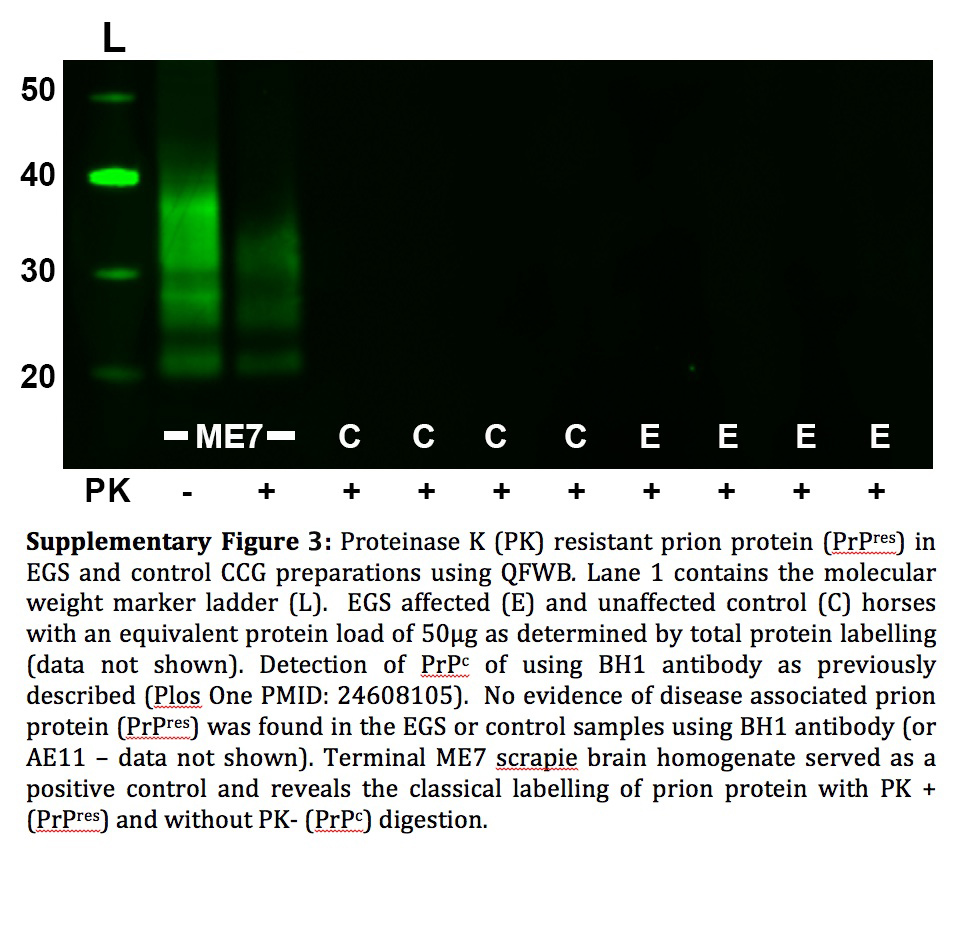

Supplement: Supplemental Data [file supp_M115.054635_mcp.M115.054635-8.jpg]
