# Supplementary material for: Proteomic Profiling of Cranial (Superior) Cervical Ganglia Reveals Beta-Amyloid and Ubiquitin Proteasome System Perturbations in an Equine Multiple System Neuropathy
Source: Mol Cell Proteomics. 2015 Sep 13;14(11):3072–86. doi: 10.1074/mcp.M115.054635 (PMC4638047; doi:10.1074/mcp.M115.054635)
Supplement: Supplemental Data [file supp_M115.054635_mcp.M115.054635-9.docx]

**McGorum et al. Supplementary discussion text**

In addition to the text in the results and discussion sections of the main manuscript the authors have included below further discussion points relevant to the experimental design and proteomic data which, although of specific interest to the neurodegenerative and equine clinical communities, would detract from the thrust of the manuscript.

***Notes on study design – Rational/limitations***

Whilst RNA based array technology may provide greater coverage of global RNAs than can be obtained for the proteome using current techniques, we would expect that global RNA alterations in response to degenerative processes, coupled with variations in individual disease progression, would provide greater “noise”, making interpretation difficult. Moreover, whilst RNA based analyses highlight what may be occurring, protein based readouts should yield more relevant/tractable information regarding active processes ([1-3](#_ENREF_1)).

Ideally, a time course study of the CCG proteome in EGS would be a natural follow up study to further clarify the nature of the neurodegenerative processes. Unfortunately this is not possible because the anatomical location of CCGs precludes collection pre-mortem, and because humane considerations necessitate timely euthanasia of horses once acute or sub-acute EGS is diagnosed. It is also important to note that while the CCG capsule was removed prior to protein extraction, CCG extracts will contain proteins derived from both neuronal and non-neuronal elements, the latter including glia, supporting cells, connective tissue, blood and blood vessels within the CCG. Consequently, some of the alterations in CCG proteome may reflect non-neuronal derived proteins, for example reflecting the presence of serum derived proteins including albumin and immunoglobulins in autonomic neurons from horses with EGS ([4](#_ENREF_4)). Regardless, we know that the most affected cells are neuronal and we can also demonstrate that whilst these neurones are undergoing degenerative processes, neuronal density is conserved between control and EGS (subacute and acute) CCG (figure 1 and supplementary figure 1). Whilst the CCG contains a mixed cell population we have previously demonstrated that iTRAQ techniques can detect pertinent alterations when as little as 10% of a cell population is affected ([1](#_ENREF_1)). In the case of EGS it appears to be neurons in the CCG which are most affected and it is therefore reasonable to assume that the majority of the alterations identified in these samples will pertain to the ongoing process of neuronal degeneration. Additionally, and, as is the case with most neurodegenerative profiling studies on predominantly neuronal tissues, it should be noted that whilst the total peptide identification in the current study is impressive, the number of proteins in an equine CCG is currently unknown. This means that although this study is an important step in our understanding of the processes underpinning the late stages of this disorder, other candidate proteins with potential roles in EGS likely remain undiscovered. However, this limitation will apply to all proteomic studies, and despite the limitations outlined above, the authors consider that the approaches taken are the most appropriate for the current study.

***Further notes on interpretation of proteomic data***

In EGS CCG, numerous proteins (n=320) were increased by greater than 20%, despite ultrastructural disruption to Golgi and ribosomes and consequent disruption to glycoprotein biosynthetic pathways being an early lesion in EGS neurodegeneration ([5](#_ENREF_5)). Increased concentration of proteins in CCG ganglion extracts could reflect increased protein synthesis in neuronal and/or non-neuronal cells in response to the unknown causal neurotoxin. Alternatively it could reflect accumulation of proteins within the degenerating perikarya due to failure of axonal transport mechanisms moving proteins from the site of synthesis in the cell body to distal parts of the nerves which lie out-with the CCG. Consistent with the latter possibility, previous studies indicate disruption of neurofilaments and microtubules in EGS ([6](#_ENREF_6)).

***Proteins involved in acute phase response;*** Several acute phase proteins were markedly increased in EGS CCG, including serum amyloid A (30.7 fold increase), serum amyloid A2 (17.4), lipopolysaccharide-binding protein (2.687), haptoglobin (2.150), fibrinogen alpha chain isoform 2 (1.952) and fibrinogen A-alpha chain (1.299). This is consistent with the systemic acute phase response previously reported in EGS (Milne et al., 1991, Copas et al., 2013). EGS CCG also associated had markedly increased osteopontin-like isoform 3 (9.685), a cytokine and matrix protein involved in neuroinflammatory and neurodegenerative processes including multiple sclerosis, Parkinson's and Alzheimer's disease. Osteopontin appears to act as a selective trigger by inducing neuronal toxicity and death in some contexts while functioning as a neuroprotectant in others ([7](#_ENREF_7)).

***Neurotransmitter associated proteins;*** EGS CCG had increased pro-neuropeptide Y (4.091) and dopamine beta-hydroxylase (2.048), consistent with previous reports ([8](#_ENREF_8)). Consistent with an increased synthesis of neuropeptides, EGS horses had increased carboxypeptidase E, isoform CRAb (2.937) which is involved in the biosynthesis of many neuropeptides ([9](#_ENREF_9)). EGS ganglia had decreased catechol O-methyltransferase (0.886), one of several enzymes that degrade catecholamines which could contribute to the elevated serum levels of dopamine, dihydroxyphenylalanine (DOPA), epinephrine and norepinephrine previously reported in EGS ([10-12](#_ENREF_10)). Similar generalised alterations in catecholamine metabolism also occur in human familial dysautonomia ([13](#_ENREF_13)). Further abnormalities in biogenic amine metabolism in EGS include elevated concentrations of plasma histamine ([14](#_ENREF_14)), skatole and indole [Pemberton; unpublished observations].

***Synaptic vesicle proteins and proteins involved in synaptic transmission;*** EGS CCG had increases in many synaptic vesicle proteins and proteins involved in synaptic transmission including SNAP-25 (2.155), synaptic vesicle membrane protein-1 (2.704), synaptotagmin 1 (2.566), protein bassoon (2.092), synapsin-1 (1.370), synaptic vesicle membrane protein VAT-1 (1.305), amphiphysin (1.315), vesicle transport through interaction with t-SNAREs homolog 1B (1.281) and syntaxin-5 (1.261). This is consistent with the previously reported increased expression of synaptophysin in autonomic neuronal perikarya in EGS ([15](#_ENREF_15)). In contrast EGS was also associated with a marked reduction in transmembrane emp24 domain-containing protein 10 (0.588), a type I membrane protein involved in vesicular protein trafficking. The latter may reflect disruption and loss of the Golgi ([5](#_ENREF_5)) since this protein is localized to the plasma membrane and Golgi cisternae ([16](#_ENREF_16)).

***VAMP fragment/botulinum toxin subunit serotype F;*** There is both historical (1920s) and modern (1990s-present) evidence to support the hypothesis that EGS is a toxico-infectious form of botulism involving *Clostridium botulinum* type-C and/or D toxin produced locally within the gastrointestinal tract ([17-22](#_ENREF_17)). Interestingly, Chain B, crystal structure of *Clostridium botulinum* neurotoxin serotype F catalytic domain with an inhibitor (Inh2) was identified in EGS CCG extracts. However, rather than this protein being a fragment of BoNT/F, it likely represents a fragment of the inhibitor, namely Vesicle-associated membrane protein (residues 27-58; ([23](#_ENREF_23))). Consistent with this possibility, attempted detection of Clostridium botulinum neurotoxin F using an undiluted rabbit polyclonal antibody (Abcam, ab27168) with QWB was unsuccessful even with a high protein load of 50 μg.

***Cytoskeletal associated proteins;*** Previous studies have identified marked alterations in the ultrastructure of the neuronal cytoskeleton and reduced expression of tubulin and phosphorylated neurofilaments NF-H and NF-L in EGS CCG ([5](#_ENREF_5)). In the current study we detected alterations in microtubule associated proteins including; MAP 1A-like (0.780), MAP 1B-like (0.800), MAP 1S-like (1.268), MAP 2-like isoform 1 (0.644), MAP 9 (0.650), MAP RP/EB family member 2 (0.742). We also detected increased expression of dematin (2.039), tryptophanyl-tRNA synthetase, cytoplasmic (1.816), palmdelphin-like (1.740), smoothelin (1.508), thymosin beta-4 (1.424), plastin-2 (1.412), profilin-2 isoform 1 (1.289) and tubulin beta-5 chain-like (1.239). EGS CCG also demonstrate decreased expression of AHNAK nucleoprotein (0.701), keratin, type I cytoskeletal 9 (0.759), alpha-internexin (0.667), kinesin light chain 2 isoform 2 (0.577).

***Proteins related to APP and its metabolism****;* In addition to increased APP, EGS CCG had increased levels of the related amyloid beta (A4) precursor-like protein 2 (3.662 ratio), cathepsin B (3.919 ratio) which degrades β-APP into harmless fragments ([24](#_ENREF_24)) and calpastatin (1.345 ratio) which is involved in the proteolysis of amyloid precursor protein ([25](#_ENREF_25)).

***Tau/MAPT;*** Upstream regulator predictions suggest that one of the potential regulators of many of the molecular consequences identified in the current study is the microtubule-associated protein Tau (Table 5). Tau is overexpressed at the protein level in EGS relative to wildtype (supplementary figure 2). However, unlike the other candidates examined we only see significantly increased expression in the acute EGS (supplementary figure 2) suggesting that this is likely a consequential rather than regulatory alteration. There is evidence for the altered expression of other candidates with the ability to regulate Tau expression in the current dataset. i.e. Cathepsin D is a known regulator of Tau degradation and it is decreased in expression (-1.758, table 3). Similarly members of the PP2A family responsible for approximatly 70% of Tau phosphatase activity (also occupying a key hub in the APP centric network (figure 7)) such as PP1 (+1.376) and PPP1R1 (+1.361) are also altered (table 5).

***Apolipoproteins;*** Several members of the apolipoprotein family were increased in EGS, including apolipoprotein A-IV (4.375), apolipoprotein A-I (1.376), apolipoprotein A-II (1.275), apolipoprotein B-100 (4.333), apolipoprotein C-II (1.291) and clusterin precursor [apolipoprotein J] (1.447), while apolipoprotein E was reduced (0.647). The family of APO proteins is quite interesting. For example, it is known that subtypes of APOE 3&4 have the ability to alter regenerative response in neurons (Comely et al 2011?), and have been associated as modulatory risk factors for the development of Alzheimer’s disease ([26](#_ENREF_26),[27](#_ENREF_27)).

***Secretogranins;*** EGS CCG also had marked increases in some of the secretogranin family of proteins, namely secretogranin-1 (8.711), secretogranin-2 precursor (3.745), and secretogranin-3 (6.966), and in neurosecretory protein VGF (7.979) which shares sequence similarities with the secretogranin / chromogranin family ([28](#_ENREF_28)) and is found in the secretory granules of subsets of neurons and endocrine cells ([29](#_ENREF_29)). The secretogranin family likely has a role in the packaging or sorting of peptide hormones and neuropeptides into secretory vesicles.

***Protein associated with GABA catabolism;*** EGS CCG had markedly reduced (0.577) 4-aminobutyrate aminotransferase, mitochondrial isoform, 1 which catabolises gamma-aminobutyric acid (GABA), a mostly inhibitory neurotransmitter in the central nervous system, into succinic semialdehyde. It is possible that increased GABA activity could account for some of the clinical features of EGS, including gastrointestinal stasis, dullness, lethargy, muscle tremors, salivation and piloerection.

***Mitochondrial proteins;*** EGS CCG had increased concentrations of mitochondrial-like citrate synthase (1.32), a mitochondrial matrix enzyme which has a key role in oxidative phosphorylation. This protein is commonly used as a quantitative mitochondrial matrix enzyme marker for the presence of intact mitochondria ([30](#_ENREF_30)). This finding is consistent with the observation that EGS CCG neurons have generally increased numbers of mitochondria which are morphologically similar to, although more slender, than normal neurons ([31](#_ENREF_31),[32](#_ENREF_32)).

**Summary:**

It is important with the application of any “mass screening” technique to make readers aware of the potential limitations of the technology and comparisons carried out in order that individuals can draw more realistic conclusions from the data generated. In addition, this document contains information on candidate proteins which will be of interest to the equine veterinary community but which would likely detract from the thrust of the main manuscript. The observations highlighted in this document linking in with existing published data serve to further demonstrate the veracity of the data produced in the current study.

**References**

1. Wishart, T. M., Rooney, T. M., Lamont, D. J., Wright, A. K., Morton, A. J., Jackson, M., Freeman, M. R., and Gillingwater, T. H. (2012) Combining comparative proteomics and molecular genetics uncovers regulators of synaptic and axonal stability and degeneration in vivo. *PLoS Genet* **8**, e1002936

2. Wishart, T. M., Huang, J. P., Murray, L. M., Lamont, D. J., Mutsaers, C. A., Ross, J., Geldsetzer, P., Ansorge, O., Talbot, K., Parson, S. H., and Gillingwater, T. H. (2010) SMN deficiency disrupts brain development in a mouse model of severe spinal muscular atrophy. *Hum Mol Genet* **19**, 4216-4228

3. Wishart, T. M., Mutsaers, C. A., Riessland, M., Reimer, M. M., Hunter, G., Hannam, M. L., Eaton, S. L., Fuller, H. R., Roche, S. L., Somers, E., Morse, R., Young, P. J., Lamont, D. J., Hammerschmidt, M., Joshi, A., Hohenstein, P., Morris, G. E., Parson, S. H., Skehel, P. A., Becker, T., Robinson, I. M., Becker, C. G., Wirth, B., and Gillingwater, T. H. (2014) Dysregulation of ubiquitin homeostasis and beta-catenin signaling promote spinal muscular atrophy. *J Clin Invest* **124**, 1821-1834

4. Griffiths, I. R., Smith, S., Kyriakides, E., and Barrie, J. M. (1994) Autonomic neurons from horses with grass sickness contain serum proteins. *Vet Rec* **135**, 90-91

5. Griffiths, I. R., Kyriakides, E., Smith, S., Howie, F., and Deary, A. W. (1993) Immunocytochemical and lectin histochemical study of neuronal lesions in autonomic ganglia of horses with grass sickness. *Equine Vet J* **25**, 446-452

6. Griffiths, I. R., Lusk, S. A., Kyriakides, E., and Smith, S. (1993) Neurones in autonomic ganglia of normal horses contain phosphorylated neurofilaments. *J Comp Pathol* **108**, 109-112

7. Carecchio, M., and Comi, C. (2011) The role of osteopontin in neurodegenerative diseases. *J Alzheimers Dis* **25**, 179-185

8. John, H. A., Creighton, A. J., and Baird, A. (2001) Thoracic sympathetic chain ganglion neuronal abnormalities that may explain some of the clinical signs of grass sickness. *Vet Rec* **148**, 180-182

9. Cawley, N. X., Wetsel, W. C., Murthy, S. R., Park, J. J., Pacak, K., and Loh, Y. P. (2012) New roles of carboxypeptidase E in endocrine and neural function and cancer. *Endocr Rev* **33**, 216-253

10. Hodson, N. P., Wright, J. A., and Hunt, J. (1986) The sympatho-adrenal system and plasma levels of adrenocorticotropic hormone, cortisol and catecholamines in equine grass sickness. *Vet Rec* **118**, 148-150

11. Hodson, N. P., Yeats, J., Wright, J. A., and Bloom, S. R. (1988) Plasma-levels of regulatory peptides (including insulin) in equine grass sickness and some other conditions. *J. Path.*  **155**, 346

12. McGorum, B. C., Wilson, R., Pirie, R. S., Mayhew, I. G., Kaur, H., and Aruoma, O. I. (2003) Systemic concentrations of antioxidants and biomarkers of macromolecular oxidative damage in horses with grass sickness. *Equine Vet J* **35**, 121-126

13. Axelrod, F. B., Goldstein, D. S., Holmes, C., Berlin, D., and Kopin, I. J. (1996) Pattern of plasma levels of catecholamines in familial dysautonomia. *Clin Auton Res* **6**, 205-209

14. Hodson, N. P., Wright, J. A., Causon, R. C., and Hunt, J. M. (1989) Plasma and tissue histamine in equine grass sickness. *J Vet Pharmacol Ther* **12**, 340-343

15. Waggett, B. E., McGorum, B. C., Shaw, D. J., Pirie, R. S., MacIntyre, N., Wernery, U., and Milne, E. M. (2010) Evaluation of synaptophysin as an immunohistochemical marker for equine grass sickness. *J Comp Pathol* **142**, 284-290

16. Stamnes, M. A., Craighead, M. W., Hoe, M. H., Lampen, N., Geromanos, S., Tempst, P., and Rothman, J. E. (1995) An integral membrane component of coatomer-coated transport vesicles defines a family of proteins involved in budding. *Proc Natl Acad Sci U S A* **92**, 8011-8015

17. Hedderson, E. J., and Newton, J. R. (2004) Prospects for vaccination against equine grass sickness. *Equine Vet J* **36**, 186-191

18. Poxton, I. R., Hunter, L. C., Brown, R., Lough, H. G., and Miller, J. K. (1997) Clostridia and equine grass sickness. *Reviews in Medical Microbiology* **8**, S49-S51

19. Hunter, L. C., Miller, J. K., and Poxton, I. R. (1999) The association of Clostridium botulinum type C with equine grass sickness: a toxicoinfection? *Equine Vet J* **31**, 492-499

20. Hunter, L. C., and Poxton, I. R. (2001) Systemic antibodies to Clostridium botulinum type C: do they protect horses from grass sickness (dysautonomia)? *Equine Vet J* **33**, 547-553

21. McCarthy, H. E., French, N. P., Edwards, G. B., Poxton, I. R., Kelly, D. F., Payne-Johnson, C. E., Miller, K., and Proudman, C. J. (2004) Equine grass sickness is associated with low antibody levels to Clostridium botulinum: a matched case-control study. *Equine Vet J* **36**, 123-129

22. Tocher, J. F., Brown, W., Tocher, J. W., and Buxton, J. B. (1923) "Grass Sickness" Investigation Report. *Vet Rec* **3**, 37-45, 75-89

23. Agarwal, R., Schmidt, J. J., Stafford, R. G., and Swaminathan, S. (2009) Mode of VAMP substrate recognition and inhibition of Clostridium botulinum neurotoxin F. *Nat Struct Mol Biol* **16**, 789-794

24. Wang, C., Sun, B., Zhou, Y., Grubb, A., and Gan, L. (2012) Cathepsin B degrades amyloid-beta in mice expressing wild-type human amyloid precursor protein. *J Biol Chem* **287**, 39834-39841

25. Higuchi, M., Iwata, N., Matsuba, Y., Takano, J., Suemoto, T., Maeda, J., Ji, B., Ono, M., Staufenbiel, M., Suhara, T., and Saido, T. C. (2012) Mechanistic involvement of the calpain-calpastatin system in Alzheimer neuropathology. *Faseb j* **26**, 1204-1217

26. Comley, L. H., Fuller, H. R., Wishart, T. M., Mutsaers, C. A., Thomson, D., Wright, A. K., Ribchester, R. R., Morris, G. E., Parson, S. H., Horsburgh, K., and Gillingwater, T. H. (2011) ApoE isoform-specific regulation of regeneration in the peripheral nervous system. *Hum Mol Genet* **20**, 2406-2421

27. Dikranian, K., Kim, J., Stewart, F. R., Levy, M. A., and Holtzman, D. M. (2012) Ultrastructural studies in APP/PS1 mice expressing human ApoE isoforms: implications for Alzheimer's disease. *Int J Clin Exp Pathol* **5**, 482-495

28. Canu, N., Possenti, R., Rinaldi, A. M., Trani, E., and Levi, A. (1997) Molecular cloning and characterization of the human VGF promoter region. *J Neurochem* **68**, 1390-1399

29. Ferri, G. L., and Possenti, R. (1996) vgf A neurotrophin-inducible gene expressed in neuroendocrine tissues. *Trends Endocrinol Metab* **7**, 233-239

30. Trounce, I. A., Kim, Y. L., Jun, A. S., and Wallace, D. C. (1996) Assessment of mitochondrial oxidative phosphorylation in patient muscle biopsies, lymphoblasts, and transmitochondrial cell lines. *Methods Enzymol* **264**, 484-509

31. Gilmour, J. S. (1975) Chromatolysis and axonal dystrophy in the autonomic nervous system in grass sickness of Equidae. . *Neuropathology and Applied Neurobiology* **1**, 39-47

32. Pollin, M. M., and Griffiths, I. R. (1992) A review of the primary dysautonomias of domestic animals. *J Comp Pathol* **106**, 99-119
